# Drainage isolation and climate change-driven population expansion shape the genetic structures of *Tuber indicum* complex in the Hengduan Mountains region

**DOI:** 10.1038/srep21811

**Published:** 2016-02-24

**Authors:** Bang Feng, Qi Zhao, Jianping Xu, Jiao Qin, Zhu L. Yang

**Affiliations:** 1 Key Laboratory for Plant Diversity and Biogeography of East Asia, Chinese Academy of Sciences, Kunming, Yunnan, China; 2 Department of Biology, McMaster University, Hamilton, Ontario, Canada; 3 University of Chinese Academy of Sciences, Beijing, China

## Abstract

The orogenesis of the Qinghai-Tibetan Plateau and the Quaternary climate changes have played key roles in driving the evolution of flora and fauna in Southwest China, but their effects on higher fungi are poorly addressed. In this study, we investigated the phylogeographic pattern of the *Tuber indicum* species complex, an economically important fungal group distributed in the Hengduan Mountains region. Our data confirmed the existence of two distinct lineages, *T. indicum* and *T. himalayense*, within this species complex. Three geographic groups (Groups W, N and C) were revealed within *T. indicum*, with Group W found in the paleo-Lancang River region, while Groups N and C corresponded to the two banks along the contemporary Jinsha River, suggesting that rivers have acted as barriers for gene flow among populations from different drainages. Historical range expansion resulted from climate changes was inferred in Group C, contributing to the observed gene flow among geographic populations within this group. Although no significant geographic structure was identified in *T. himalayense*, evidence of drainage isolation for this species was also detected. Our findings demonstrate that both topographic changes and Quaternary climate oscillations have played important roles in driving the genetic structures of the *T. indicum* species complex.

Inferring the driving forces for the microevolution of organisms has been one of the central topics in phylogeography^1^. The Himalaya-Hengduan Mountains region, one of the global biodiversity hotspots located in Southwest China^2^,^3^, has attracted broad attention from researchers for several decades. A great deal of studies have identified the dramatic topographic changes triggered by the rapid uplifting of the Qinghai-Tibetan Plateau (QTP) and the Quaternary climate oscillations as two key evolutionary factors that drive the patterns of genetic diversity and genetic structures of plants and animals in the QTP and its adjacent regions^4^.

The rapid uplifting of the QTP has formed the complex mountain systems in Southwest China, which act as significant physical barriers for gene flow of many organisms, leading to their strong population differentiations and even rapid speciation^5^,^6^,^7^. Furthermore, this important tectonic event has greatly reshaped the river systems in Southwest China. Studies have shown that several plants and animals have reacted to the drainage changes differently during their evolutionary processes. For example, the genetic structures of several river valleys-associated plants, such as *Terminalia franchetii* and *Buddleja crispa*, have been significantly influenced by the paleodrainage systems but not by the contemporary drainages^8^,^9^,^10^. In contrast, the phylogeographic pattern of *Nanorana yunnanensis* has been shaped by both paleo- and contemporary drainages^11^.

In addition to the geological events, the global cyclic cooling-warming events during the Quaternary period can also significantly impact the evolutionary histories of organisms^12^,^13^. Generally, populations tend to contract to southern or *in situ* refugia during glaciations and then expand to northern or adjacent regions as glaciations retreat. In Southwest China, several research works have identified the southern or *in situ* refugia in the QTP for different plants and animals^14^,^15^,^16^,^17^,^18^,^19^,^20^. However, there is also evidence for the persistent and stable populations of *Quasipaa boulengeri* in the mountains of Southwest China through the glacial cycling^6^.

The combined historical tectonic and climatic changes can play different roles in driving the phylogeographic patterns of different organisms. In Southwest China, most of the available phylogeographic studies have focused on plants and animals. The evolutionary histories of higher fungi, as well as their relationships to the geological and climate changes, are relatively poorly understood. The only available phylogeographic study on an ectomycorrhizal fungus in this area, *Tricholoma matsutake*, indicated that the contemporary river systems could act as barriers for gene flow among geographic populations^21^.

The *Tuber indicum* species complex, commonly known as the Chinese black truffle, is a group of economically important ectomycorrhizal fungi. It contains two distinctive lineages^22^,^23^,^24^,^25^,^26^,^27^, both of which form symbiotic relationships with several plant families, such as Pinaceae and Fagaceae^23^,^26^,^27^. This species complex is mostly restricted to the Hengduan Mountains region in Southwest China, an area with spectacular rugged mountain ranges and deep gorges carved by the “Three Rivers”, the Jinsha, Lancang and Nu Rivers (Fig. 1b), which are the main upstream tributaries of the Yangtze, Mekong and Salween Rivers, respectively. This region features extreme changes in elevation over very short horizontal distances. The mountains and rivers can act as physical barriers for the dispersal of these truffles. Like other hypogeal fungi, the *T. indicum* species complex requires small rodents or other mycophagists to help disperse their spores^28^. Because these barriers can limit the dispersal of the carrier animals of truffles, we hypothesize that the historical tectonic events have significant influence on the genetic structure of the *T. indicum* species complex. Meanwhile, this species complex is believed to be cold-tolerant, as it can produce mature ascocarps in winter. We are thus interested to test if the Quaternary climate oscillations have impacts on its evolutionary history.

In this study, we sampled extensively and investigated the phylogeographic patterns of the *T. indicum* species complex from the Hengduan Mountains region by analyzing the sequences of four DNA fragments (Tic1–3, three fragments randomly selected from the genome, and part of the mini–chromosome maintenance complex component, *mcm*7). We estimated the demographic history of this species complex by using species distribution modeling. We aimed to (i) reveal the genetic structure of the *T. indicum* species complex, (ii) reconstruct its historical demography, and (iii) explore the relative roles of geological and climate changes in shaping the phylogeographic structures of this species complex.

## Results

### Genetic structure and diversity

**Figure 1:**
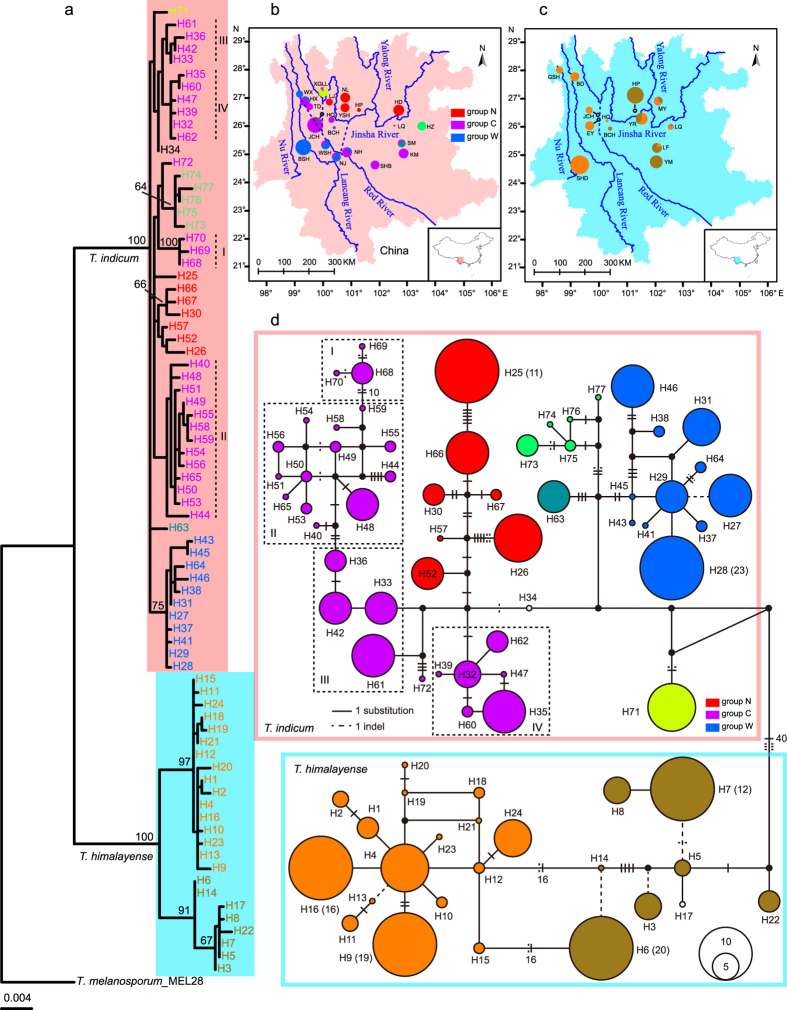
Collection sites for the *Tuber indicum* species complex and distribution patterns of geographic groups within this species complex. (**a**) shows the phylogenetic tree generated from the 77 haplotypes of the *T. indicum* species complex, with two species, *T. indicum* and *T. himalayense*, identified. (**b**,**c**) display the distribution patterns of the geographic groups identified within *T. indicum* and *T. himalayense* respectively. (**d**) gives the haplotype network generated from the 77 haplotypes of the *T. indicum* species complex. All maps were drawn using ArcGIS V.10.2 (ESRI, CA, USA) and then improved in Adobe Illustrator CC (Adobe Systems Inc., CA, USA).

DNA was isolated from 327 ascocarps collected from 30 localities and was amplified with primer pairs targeting the Tic1, Tic2, Tic3 and *mcm*7 markers. The aligned sequences of Tic1, Tic2, Tic3 and *mcm*7 were 429, 500, 500 and 536 bp in length, respectively, and they did not show any heterozygosity within any single ascocarp. In total 77 haplotypes were detected by using the combined four-gene matrix. In the phylogenetic tree inferred using the Maximum Likelihood method (Fig. 1a), the 77 haplotypes clustered into two well-supported lineages, one including 24 haplotypes (H1–H24) and the other consisting of 53 haplotypes (H25–H77). The median-joining haplotype network (Fig. 1d) revealed two clades corresponding to the two lineages illustrated by the phylogenetic tree. The analysis of molecular variance (AMOVA) revealed that 75.59% of the total observed genetic variations were partitioned to between the two lineages, 18.11% to among geographic populations within the two lineages, and 6.29% to within individual geographic populations (Table 1). Interestingly, the two lineages corresponded to the two previously defined species of the *T. indicum* species complex, *T. indicum* and *T. himalayense*^24^,^26^. We thus named them as *T. indicum* and *T. himalayense* respectively (see also Discussion). Five localities (HQ, BCH, LQ, HP and JCH) harbored haplotypes from both lineages. For the remaining 25 localities, GSH, SHD, BD, EY, YR, MY, YM and LF possessed haplotypes from *T. himalayense*, while the rest localities harbored haplotypes from *T. indicum*.

Significant geographic structure was observed within each of the two lineages. Within *T. indicum*, three geographic groups could be identified by the phylogenetic tree and/or haplotype network: (i) haplotypes from four western populations (WX, BSH, WSH and NJ, named as Group W) formed a subclade in the phylogenetic tree (Fig. 1a,b), which was supported by the haplotype network (blue colored; Fig. 1b,d); (ii) haplotypes from five northern populations (LJ, YSH, NL, HP and HD, named as Group N) clustered together in the haplotype network (red colored; Fig. 1b,d); and (iii) haplotypes from seven populations in the central region (HX, TD, JCH, HQ, NH, SHB and KM, named as Group C) formed two closely related clusters in the haplotype network (pink colored, Fig. 1b,d). The remaining three populations (HZ, SM and XGLL) harbored haplotypes that did not cluster into any of these three groups. For *T. himalayense*, two well-supported subclades could be inferred from both the phylogenetic tree and the haplotype network (Fig. 1a,c,d), with the first subclade mainly from eastern populations (HP, LF and YM) and the second mainly from western populations (GSH, SHD, BD, JCH and EY). Four populations in the eastern region (MY, YR, LQ and LF) harbored haplotypes in both subclades.

Molecular genetic diversity indices, including haplotype diversity (Hd), number of private haplotypes and nucleotide diversity (π), for each population of *T. indicum* and *T. himalayense* are summarized in Table 2. Sixty-three out of 77 haplotypes were restricted to certain populations, with 21 and 42 private haplotypes from *T. himalayense* and *T. indicum* respectively. In *T. indicum*, Hd ranged from 0 to 0.937, and π from 0 to 0.00419, while for *T. himalayense*, Hd ranged from 0 to 0.933, and π from 0 to 0.00820.

### Demographic history

**Figure 2:**
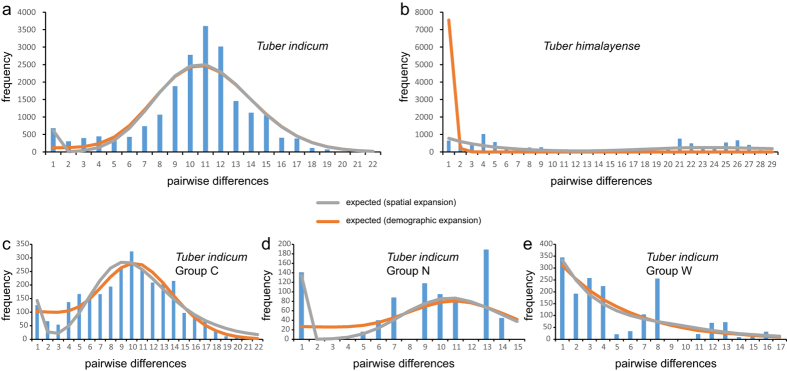
Population demography of the *Tuber indicum* species complex estimated by mismatch distribution analyses. (**a**) and (**b**) show the demographies of *T. indicum* and *T. himalayense* respectively, while (**c**), (**d**) and (**e**) show the demographies of Groups C, N and W of *T. indicum* respectively.

Tajima’s D and Fu’s Fs values were positive in *T. himalayense* but were negative at the species level and in Group C of *T. indicum*. The negative Fu’s Fs value at the species level of *T. indicum* was statistically significant (Table 3). In addition, unimodal mismatch distributions were observed in *T. indicum* and Group C (Fig. 2). However, SSD and HRI were statistically significant at the species level but not in Group C (Table 3). Thus, models of spatial and demographic expansion could not be significantly rejected for Group C.

### Species distribution modeling (SDM)

**Figure 3:**
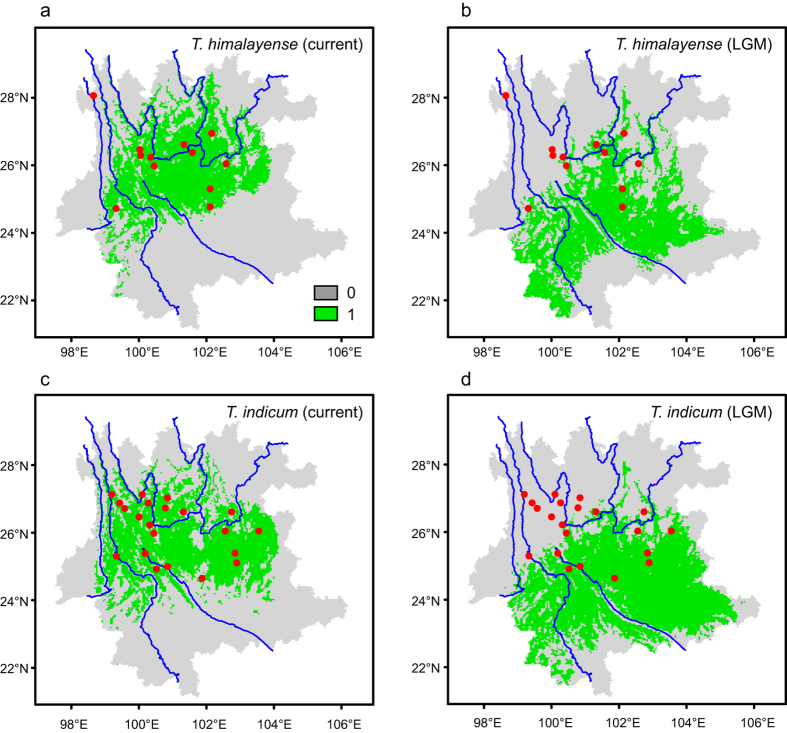
Suitable habitats for the *Tuber indicum* species complex under current and the Last Glacial Maximum (LGM) climatic conditions predicted by species distribution models. 0 and 1 indicate unsuitable and suitable habitats respectively. Red cycles show the localities where strains of each species were collected. (**a, c**) show the estimated distribution ranges of *T. himalayense* and *T. indicum* under current climatic condition. (**b, d**) show the estimated distribution ranges of *T. himalayense* and *T. indicum* under the LGM climatic condition. All maps were generated using ArcGIS V.10.2 (ESRI, CA, USA) and then improved in Adobe Illustrator CC (Adobe Systems Inc., CA, USA).

SDMs were reconstructed to predict the potential suitable habitat for *T. himalayense* and *T. indicum* under current and the Last Glacial Maximum (LGM) climatic conditions. For *T. himalayense*, the average AUC (area under the receiver operating characteristic curve) value was 0.882 with a standard deviation of 0.034, while for *T. indicum*, the average AUC and the standard deviation were 0.931 and 0.022 respectively. These statistics indicated that the distribution prediction for each species was far better than a random prediction. For both species, the predicted distribution under the LGM (Fig. 3b,d) showed a shift into southern and southeastern regions while compared with the predicted current distribution (Fig. 3a,c).

## Discussion

Prior to this study, high morphological and molecular polymorphisms have been observed in several studies concerning the *T. indicum* species complex^24^,^26^,^27^. Two lineages have been identified in this species complex by using sequence data of different gene markers (i.e., ITS, nrLSU, *tef*1 and β-*tubulin*)^22^,^23^,^24^,^25^,^26^,^27^. However, whether these two lineages should be treated as two species or as two geographical ecotypes of a single species has remained controversial. In this study, extensive sampling of the species complex confirmed the existence of two divergent lineages (Fig. 1a,d). One of the important reasons that the two lineages have been considered as two geographic ecotypes of the same species was that “a west–east sinuous line between the two groups” has been observed^27^. However, different from previous observations, our data showed that these two lineages were sympatrically distributed in several localities, with five (JCH, HQ, BCH, LQ and HP) out of 30 collection sites harboring both lineages (Fig. 1b,c, Supplementary Fig. S1). Our result is similar to the observation that samples from Huize (HZ) could be divided into two lineages^24^,^26^. The sympatric distribution of these two distinctively divergent lineages indicates that there is reproductive isolation between them in nature. Indeed, it has been revealed that “sequence variations and rearrangements in both coding and non-coding regions” could be detected in the mating type locus of the different ITS classes (which are corresponding to the two lineages identified in this study) within the *T. indicum* species complex^29^. Given the fact that the *T. indicum* species complex has an outcrossing reproductive mode^29^, the polymorphisms at the mating type locus are also consistent with reproductive isolation between these two lineages. Therefore, our results support these two lineages as two separate species, *T. indicum and T. himalayense*, as proposed by several researchers before^24^,^26^.

In the past few years, several studies have indicated strong associations between the paleo-drainage systems and current genetic patterns of flora living in river valleys (like *Terminalia franchetii* and *Buddleja crispa*)^8^,^9^,^10^ and aquatic/hygrophilous fauna (like *Nanorana yunnanensis* and *Hemibagrus macropterus*)^11^,^30^. Similar to these observations, our study revealed that three geographical groups of *T. indicum* had strong correlations with paleo- or contemporary drainage systems (Fig. 1b): Group W consisted of samples from the Lancang River (WX and BSH) and the upper Red River (WSH and NJ); Group N possessed samples mainly from the northern bank of the Jinsha River (NL, YSH, HP and HP), with one exception, LJ, which is located on the southern bank of the Jinsha River; Group C contained samples mainly from the southern bank of the Jinsha River (HX, TD, JCH, HQ, NH, SHB and SM). Interestingly, the distribution of *T. indicum* is not strongly associated with rivers. Instead, it occurs mainly in mountains with moderate elevations (1800–2500 m) (Supplementary Table S1). Because the *T. indicum* species complex form only underground ascocarps, its spores can not spread by wind but require the help of small rodents or other mycophagists. At present, there is no direct evidence that mycophagists such as mice can cross large rivers. Thus, the large rivers in Southwest China might have acted as the barriers for the movements of mycophagists and have indirectly restricted the gene exchanges of hypogeal mushrooms between two banks of the rivers.

The geographical structure of *T. indicum* could have resulted from the evolution of drainage systems in the Hengduan Mountains region, and from its distribution range shift driven by historical climate fluctuations. According to available geomorphological evidence, paleo-drainages in Southwest China differed significantly from contemporary hydrological systems: the upper regions of the Mekong, Salween and Yangtze Rivers have historically been tributaries to the paleo-Red River system, while the modern Jinsha River was formed by the captures and reversals of several tributaries (like the Dadu, Jialing and Yalong Rivers)^31^. Group W was the only group whose distribution showed correlations with the paleo-drainage. Populations within this group are mainly located in the west of the paleo-Red River (Fig. 1b). The drainage basins in this region have experienced limited change since the early Pliocene^32^. The river capture events of the Mekong (Lancang) River have been located in its upstream in southeastern Tibet^31^, contributing to the long-time stability of the distribution of Group W. Concerning Groups C and N, their distribution patterns showed obvious concordance with the modern Jinsha River. However, several signals of paleo-drainage influence could be detected in both of them. For example, five populations (HD, NL, HP, YSH and LJ) that clustered in Group N showed significant sequence variations (Fig. 1d), indicating that historical isolations could have occurred among them. In Group C, three subgroups, I, II and III, could be assigned to different paleo-drainage basins (as indicated by the dash lines in Fig. 1b): subgroup I possessed haplotypes from HQ, subgroup II comprised samples mainly from the middle ground of the Jinsha River and the Lancang River (JCH, TD and HX), while subgroup III contained strains from the contact zone between the Jinsha River and the Red River (NH, SHB and KM). However, the limited divergence among haplotypes within these subgroups, together with the fact that a fourth subgroup (IV) consisted of strains from JCH, HX, SHB, KM and SM, suggested that gene flows among different populations within Group C have likely occurred after the formation of the modern Jinsha River. Interestingly, the species distribution modeling showed that *T. indicum* populations have retreated into southern regions during the LGM (Fig. 3d) and then expanded its distribution ranges to northern regions (Fig. 3c) after the glacial time. Furthermore, the mismatch distribution estimation could not reject the hypothesis of demographic and spatial expansions of Group C (Fig. 2 and Table 3). The range shift and expansion thus could have accelerated the admixture of these subgroups, facilitating the gene flows among different populations in the southern bank of the Jinsha River.

Groups W, C and N within *T. indicum* mostly comprised reciprocally exclusive haplotypes. However, 11 haplotypes were distributed outside of their expected geographic ranges (Fig. 1b). These included five haplotypes in Group W (H37, H38, H43, H45 and H64) from four populations of the central region (HX, JCH, BCH and NH), four haplotypes in Group C (H33, H39, H40 and H47) from two populations of the western region (NJ and WSH), one haplotype (H49) in Group C from one population of the northern region (LJ), and one haplotype in Group N (H57) from one population of the central region (JCH). The populations harboring these outlier haplotypes were mainly located at the contact zones of different geographic groups and possessed higher nucleotide diversities (0.0017 to 0.0042; Table 2) than other populations. These results strongly suggest that secondary contacts have occurred between groups previously isolated by paleo-drainages after the rearrangements of these river systems.

*Tuber himalayense* and *T. indicum* are sympatrically distributed. Therefore, they would have experienced similar evolutionary history. Surprisingly, we failed to reveal significant geographic structure in *T. himalayense* as found in *T. indicum*. Compared with *T. indicum*, *T. himalayense* showed relatively limited sequence variations in the four DNA fragments used in this study, except for the four populations (MY, YR, LQ, and LF) that harbored both types of the strongly divergent Tic3 sequences. This indicates that these two species have likely evolved at different rates. As a result, the DNA sequences, which are informative to infer the phylogeoraphic structure of *T. indicum*, appeared to be less useful to study *T. himalayense*. Despite the potential shortcoming, our analyses showed that rivers have acted as barriers for the gene flow among populations within *T. himalayense*. The two genotype groups of this species (Fig. 1a,d), which were introduced by the two strongly divergent types of Tic3 sequences (Supplementary Fig. S2), could be roughly divided by the paleo- Red River as eastern and western groups (as shown in Fig. 1c), although four populations (MY, YR, LQ and LF) in the eastern region also harbored haplotypes from the western group, suggesting that paleo- Red River would have blocked the gene flow of type II (Supplementary Fig. S2) of Tic3 from entering the western region. Other rivers were also found as potential barriers for gene flow among the western populations. For instance, GSH and SHD, two populations located on the western and eastern banks of the Nu River respectively, had their own private haplotypes (Fig. 1d). At present, the reasons for why Tic3 showed a different phylogenetic pattern are not known. However, both incomplete lineage sorting and recent introgression with *T. indicum* could have contributed to the observations.

We would like to note that although the *T. indicum* species complex has been proven to be heterothallic^29^, we failed to identify heterozygosity within any single ascocarp. The result is consistent with the observation that the gleba (major source for DNA isolated in truffle ascocarps) of *T. melanosporum*, a close relative of the *T. indicum* species complex, is maternally inherited^33^,^34^. Whether markers from paternal and maternal parents of *T. indicum* and *T. himalayense* would show different phylogeographic patterns remains to be investigated.

Apart from inferring the evolutionary history of the *T. indicum* species complex, our study suggested certain guidelines for the conservation of *T. indicum* and *T. himalayense*. During the past two decades, the local mushroom hunters and traders in Southwest China have enjoyed the economic benefits of *T. indicum* and *T. himalayense*. Unfortunately, it has led to destructive over-harvesting of these two fungal species^35^. The early hunting for truffles in Yunnan and Sichuan Provinces begins around midsummer as young ascocarps are considered to have better taste. From then on, the same truffle “burn” may be dug repeatedly until the deep winter. This practice for truffle harvesting can destroy the underground mycelia systems and reduce the production of mature ascospores. Our study identified low intrapopulation genetic diversity within both *T. indicum* and *T. himalayense* (Table 2). Frequently, the genetic diversity is even lower in areas where the truffle resources have been harvested and traded for a relatively longer time, such as MY and HD in Sichuan Province, as well as YSH, NH and HZ in Yunnan Province, indicating that the truffle resources in these regions may be declining very fast. Thus, priorities for protecting truffle resources should be given to the areas that have a relatively shorter harvesting history and harbor higher intra-population genetic diversities, such as JCH, TD, HX, WSH and EY. To avoid habitat destruction, some gentle however efficient mushroom harvesting methods (for example, training truffle dogs) should be employed.

In summary, our phylogeographic study on the *T. indicum* species complex provided the first insights into the influence of tectonic and climatic events on the evolution of hypogeal fungi in Southwest China. We confirmed the existence of two distinct lineages, *T. indicum* and *T. himalayense*, within this species complex. Both paleo- and contemporary drainages have shaped the phylogeographic structure of *T. indicum*, while historical climatic events during the Quaternary have facilitated the gene flow among populations along the southern bank of the Jinsha River. Our study also provides essential genetic background for the conservation of this economically important fungal group.

## Materials and Methods

### Sample collection

In total 327 ascocarps of the *T. indicum* species complex were collected from 30 localities distributed in different counties of Yunnan and Sichuan Provinces during 2007–2011 (Supplementary Table S1) with the help of local mushroom hunters. The collecting sites with the elevations ranging from 1200–2500 m covered the known east-west and south-north boundaries of this species complex in Mainland China. Samples from different drainages were collected to detect the possible influences of river systems on the genetic structures of this species complex. Voucher specimens are deposited in the Herbarium of Cryptogams, Kunming Institute of Botany, the Chinese Academy of Sciences.

### Selection for gene markers

To select gene markers, we firstly constructed a mini genomic library of the *T. indicum* species complex using one strain collected from HD. Procedures for the library construction were as follows: total DNA was isolated using an EZNA fungal DNA kit (Omega, USA) and then digested with restriction enzyme *afa*I. The obtained fragments were then ligated to a pMD18-T vector (Takara, Japan) and transformed into *E. coli* strains. Fragments having length ranges about 500–800 bp were randomly selected for sequencing and primer pairs were designed based on the sequenced fragments using the online software Primer3^36^. Another strain collected from YR was amplified with these primer pairs and sequenced. Three fragments with different polymorphisms were selected based on the comparison of sequences from the two strains from HD and YR. These three gene markers were named here as Tic1, Tic2 and Tic3. Furthermore, *mcm*7, which was proven to be valuable for both lower and higher level phylogenetic analyses within the Ascomycota^37^, was chosen as a conserved gene marker. Detailed information about the positions of these four fragments in the genome of *T. melanosporum*, a close relative of the *T. indicum* species complex, and the sequences of the primer pairs designed for these gene markers are listed in Supplementary Table S2.

### DNA extraction, amplification and sequencing

Total DNA of each sample was isolated using the CTAB method^38^. Fragments of Tic1, Tic2, Tic3 and *mcm*7 were amplified with the primer pairs listed in Supplementary Table S2. The amplifications were conducted on an ABI 2720 Thermal Cycler (Applied Biosystems, Foster City, CA, USA) using the following program: pre-denaturation at 94 ^o^C for 3 min, then followed by 35 cycles of denaturation at 94 ^o^C for 1 min, annealing at 50–53 ^o^C for 1 min and elongation at 72 ^o^C for 1 min, afterward, a final elongation at 72 ^o^C for 8 min was followed. The obtained products were purified with a Gel Extraction & PCR Purification Combo Kit (Bioteke, Beijing, China) and then sequenced on an ABI-3730-XL DNA Analyzer (Applied Biosystems, Foster City, CA, USA) using the same primer pairs as used in amplifications or using the primer designed for sequencing. All sequencing with forward primers obtained very clear chromatograms. As Tic1, Tic2 and *mcm*7 showed clear but lower level of polymorphisms than Tic3, we thus didn’t sequence these three fragments with their reverse primers. For Tic3, bi-directional sequencing was conducted for each strain to ensure the accuracy of sequences obtained.

### DNA sequence alignment

Sequences of Tic3 gotten from both directions were assembled with SeqMan compiled in the Lasegene software suite. For the other three fragments, sequences were carefully checked with the chromatograms. Sequences for each fragment were aligned with ClustalX^39^ and manually edited on Bioedit v7.2.3^40^ while necessary. Identical haplotypes for each gene marker were collapsed using DnaSP5^41^ and were submitted to GenBank with accession numbers from KJ653273–KJ653358. Subsequently, four matrices of Tic1, Tic2, Tic3 and *mcm*7 were concatenated to form a four-gene matrix by using Phyutility v2.6^42^ to conduct the followed phylogenetic and phylogeographic analyses.

### Phylogenetic analysis and Genealogical reconstruction

Identical haplotypes for the four-gene matrix were collapsed using DnaSP5^41^. Phylogenetic relationships among these haplotypes were then inferred by phylogenetic analysis on RAxML v7.2.6^43^, with *T. melanosporum* chosen as outgroup. All parameters in the phylogenetic analysis were kept as default setting, and statistical support values were obtained using a nonparametric bootstrapping with 1000 replicates. Furthermore, the relationships among haplotypes were inferred using median-joining haplotype network reconstruction^44^ implemented in the software NETWORK v4.6.11 (http://www.fluxus-engineering.com). The maximum parsimony option^45^ was employed to clean up the networks. In this analysis, all indels were recoded as single mutation events by using GapCoder^46^ and all indels longer than 1 bp were double weighted as suggested by the manual of the software.

### Molecular diversity and genetic structure

Genetic diversity for each population, including haplotype diversity (Hd) and nucleotide diversity (π), were inferred using Arlequin v3.5^47^. Analysis of molecular variance (AMOVA)^48^ implemented in Arlequin was employed to demonstrate population structure of the *T. indicum* species complex with significance assessed by 10000 permutations. Populations were grouped according to the two lineages identified in the phylogenetic analysis and the genealogical reconstruction (see Results).

### Population demography

Two statistical strategies, neutrality test and mismatch distribution analysis, were carried out to examine the historical population dynamics of the two species and the three main haplotype groups within *T. indicum*. In neutrality test, a significant negative Tajima’s D^49^ or Fu’s Fs^50^ value might indicate recent demographic or spatial expansion. In mismatch distribution analysis, a ragged, multimodal mismatch distribution is expected with a long-term constant population size, while a smooth and unimodal distribution would suggest a sudden demographic or spatial expansion^51^. The validity of expansion model was evaluated with the sum of squared deviations (SSD) and the raggedness index (HRI) of the observed distributions by conducting 1000 bootstrap replicates. Non-significant SSD and HRI would suggest demographic or spatial expansion. Both neutrality tests and mismatch distribution analyses were conducted in Arlequin.

### Species distribution modeling

The species distribution models were generated using the maximum entropy machine-learning algorithm^52^ as implemented in the program Maxent v3.3.3k. We used the collection sites in this study as the occurrence data for the modeling. A set of 19 bioclimatic variables for both current (1950–2000) and the Last Glacial Maximum (LGM, 21000 years before present) conditions were downloaded from the WorldClim v1.4 database (http://www.worldclim.org) with a resolution of 2.5 arc-minutes. Layers of the LGM conditions were obtained by the community climate system model (CCSM). To minimize overfitting of the SDMs led by strong correlations between pairs of variables, we assessed the correlation between each pair of the 19 bioclimatic variables by using SDMtoolbox v1.1c^53^. All pairs showed correlation lower than 0.8 and thus all the variables were included in the modeling.

SDMs were constructed using bootstrap strategy (100 replicates), with 25% of occurrence points defined as test data. The maximum iteration was set to 5000 and the other parameters were set to the default values. Model performance was assessed through comparison of the area under the receiver operating characteristic curve (AUC) values for training and test data. An AUC of 0.5 indicates a random prediction of presence and absence, while an AUC of 1 represents a perfect simulation. Subsequently, we adopted the “minimum training presence logistic threshold” as the cutoff value to divide the background into suitable and unsuitable habitats by using ArcGIS v10.2.

## Additional Information

**How to cite this article**: Feng, B. *et al*. Drainage isolation and climate change-driven population expansion shape the genetic structures of *Tuber indicum* complex in the Hengduan Mountains region. *Sci. Rep*. **6**, 21811; doi: 10.1038/srep21811 (2016).

## Supplementary Material

Supplementary Information

## Figures and Tables

**Table 1:** Results of the analysis of molecular variance (AMOVA) for four-gene sequence data of the *Tuber indicum* species complex.

Source of variation	Degree of freedom	Sum of squares	Variance components	Percentage of variation	Index fixation
Among groups	1	2465.805	15.65706	75.59	
Among populations within groups	32	1173.919	3.75174	18.11	
Within populations	293	381.811	1.30311	6.29	
Total	326	4021.535	20.71191		0.937 (p < 0.01)

**Table 2:** Summary of haplotype information and genetic diversity of each population within *Tuber indicum* and *T. himalayense*.

Code		Genetic diversity		
	Haplotypes (numbers of individuals)	Hp	Hd	π (×10^−2^)
*Tuber himalayense*				
YR	H1(4), H2(3), H3(5)	3	0.712 ± 0.069	0.820 ± 0.446
MY	H4(6), H5(3)	1	0.500 ± 0.128	0.641 ± 0.365
YM	H6(13)	0	0	0
HP	H7(12), H8(5)	2	0.441 ± 0.098	0.026 ± 0.027
SHD	H9(19)	1	0	0
EY	H4(1), H10(2), H11(3), H12(2), H13(1)	4	0.861 ± 0.087	0.098 ± 0.072
LF	H6(7), H14(1), H15(2)	2	0.511 ± 0.164	0.364 ± 0.213
BD	H16(9)	0	0	0
JCH	H16(7)	0	0	0
LQ	H17(1), H18(2), H19(1), H20(1), H21(1)	5	0.933 ± 0.122	0.524 ± 0.325
BCH	H22(4)	1	0	0
HQ	H4(2), H23(1)	1	0.667 ± 0.314	0.039 ± 0.048
GSH	H24(7)	1	0	0
*Tuber indicum*				
HD	H25(11)	1	0	0
YSH	H26(9)	1	0	0
BSH	H27(8), H28(19), H29(5)	1	0.579 ± 0.069	0.052 ± 0.041
HP	H30(4)	1	0	0
WX	H31(7)	1	0	0
SHB	H32(3), H33(5), H34(1)	1	0.639 ± 0.126	0.149 ± 0.100
HX	H32(1), H35(4), H36(3), H37(1), H38(1)	0	0.800 ± 0.100	0.331 ± 0.196
WSH	H28(4), H29(1), H38(1), H39(1), H40(1), H41(1)	3	0.833 ± 0.127	0.381 ± 0.226
NH	H42(6), H43(1), H44(1), H45(1)	4	0.583 ± 0.183	0.397 ± 0.234
NJ	H33(1), H46(8), H47(1)	2	0.378 ± 0.181	0.224 ± 0.139
TD	H48(3), H49(1), H50(2), H51(1)	2	0.810 ± 0.130	0.166 ± 0.114
LJ	H49(1), H52(6)	1	0.286 ± 0.196	0.166 ± 0.114
LQ	H53(2)	1	0	0
JCH	H32(1), H35(4), H36(1), H37(1), H48(3), H54(1), H55(2), H56(2), H57(1), H58(1), H59(1), H60(2)	7	0.937 ± 0.033	0.419 ± 0.229
KM	H61(2), H62(8)	1	0.356 ± 0.159	0.083 ± 0.062
SM	H62(2), H63(6)	1	0.427 ± 0.169	0.224 ± 0.143
BCH	H64(2), H65(1)	2	0.667 ± 0.314	0.388 ± 0.314
NL	H66(8), H67(2)	2	0.356 ± 0.159	0.083 ± 0.062
HQ	H68(4), H69(1), H70(1)	3	0.600 ± 0.215	0.097 ± 0.075
XGLL	H71(9), H72(1)	2	0.200 ± 0.154	0.105 ± 0.074
HZ	H73(4), H74(1), H75(2), H76(1), H77(1)	5	0.806 ± 0.120	0.172 ± 0.112

Haplotypes shared by different populations are underlined. Abbreviations: Hp, number of private haplotypes; Hd, haplotype diversity; π, nucleotide diversity.

**Table 3:** Summary of results from neutrality tests and mismatch distribution analyses on *Tuber indicum* and *T. himalayense*, as well as three haplotype groups within *T. indicum*.

Grouping	Tajima’s D	Fu’s Fs	Demographic expansion		Spatial expansion	
			SSD	HRI	SSD	HRI
*Tuber indicum*						
all	−0.817	−13.542**	0.009**	0.014**	0.009	0.014
north	1.079	7.167	0.065**	0.166**	0.043	0.166
west	−0.806	0.572	0.027	0.063	0.027	0.063
central	−0.421	−5.515	0.002	0.005	0.004	0.005
*T. himalayense*						
all	2.157	3.041	0.903**	0.025	0.023	0.025

^**^p < 0.01.
