# Supplementary material for: Drainage isolation and climate change-driven population expansion shape the genetic structures of Tuber indicum complex in the Hengduan Mountains region
Source: Sci Rep. 2016 Feb 24;6:21811. doi: 10.1038/srep21811 (PMC4764814; doi:10.1038/srep21811)
Supplement: Supplementary Information [file srep21811-s1.pdf]

# Drainage isolation and climate change-driven population expansion shape the genetic structures of *Tuber indicum* complex in the Hengduan Mountains region

Bang Feng[1] Qi Zhao[1] Jianping Xu[2] Jiao Qin[1,3] Zhu L. Yang[1*]

1 Key Laboratory for Plant Diversity and Biogeography of East Asia, Chinese Academy of Sciences, Kunming, Yunnan, China

2 Department of Biology, McMaster University, Hamilton, Ontario, Canada

3 University of Chinese Academy of Sciences, Beijing, China

*Correspondence: Zhu L. Yang, Key Laboratory for Plant Diversity and Biogeography of East Asia, Kunming Institute of Botany, Chinese Academy of Sciences, Kunming 650201, Yunnan, China, fungi@mail.kib.ac.cn

Table S1 Summary of sampling sites in Yunnan (YN) and Sichuan (SCH) Provinces for *Tuber indicum* complex.

| **Locality** | **Code** | **N** | **Drainage** | **Long.** | **Lat.** | **Alt. (m)** | **Col. Year** |
|---|---|---|---|---|---|---|---|
| ***Tuber himalayense*** | | | | | | | |
| Bingzhongluo,Gongshan Co., YN | GSH | 7 | West of Upper Nu River | 98.61 °E | 28.03 °N | 1719 | 2007 |
| Bailang,Shidian Co., YN | SHD | 19 | East of Lower Nu River | 99.25 °E | 24.67 °N | 2021 | 2009 |
| Badi, Weixi Co., YN | BD | 9 | East of Lancang River | 99.01 °E | 27.87 °N | 1801 | 2009 |
| Jianchuan Co., YN | JCH | 7 | South of Middle Jinsha River | 99.92 °E | 26.45 °N | 2234 | 2009 |
| Niujie, Eryuan Co., YN | EY | 9 | South of Middle Jinsha River | 99.99 °E | 26.25 °N | 2101 | 2009 |
| Songgui, Heqing Co., YN | HQ | 3 | South of Middle Jinsha River | 100.20 °E | 26.35 °N | 1944 | 2011 |
| Jizushan, Binchuan Co., YN | BCH | 4 | South of Middle Jinsha River | 100.40 °E | 25.94 °N | 1860 | 2011 |
| Rongjiang, Huaping Co., YN | HP | 17 | North of Middle Jinsha River | 101.28 °E | 26.57 °N | 1458 | 2009 |
| Yongxing, Yongren Co., YN | YR | 12 | South of Middle Jinsha River | 101.53 °E | 26.34 °N | 1732 | 2007 |
| Panlian, Miyi Co., SCH | MY | 9 | North of Middle Jinsha River | 102.13 °E | 26.81 °N | 1546 | 2007 |
| Tongchang, Yimen Co., YN | YM | 13 | South of Middle Jinsha River | 102.04 °E | 24.73 °N | 2152 | 2007 |
| Jinshan, Lufeng Co., YN | LF | 10 | South of Middle Jinsha River | 102.06 °E | 25.25 °N | 1630 | 2007 |
| Sayingpan, Luquan Co., YN | LQ | 6 | South of Middle Jinsha River | 102.52 °E | 25.98 °N | 2197 | 2009 |
| ***T. indicum*** | | | | | | | |
| Baijixun, Weixi Co., YN | WX | 7 | East of Lancang River | 99.17 °E | 27.09 °N | 1606 | 2009 |
| Shuizhai, Baoshan,YN | BSH | 32 | West of Lancang River | 99.30 °E | 25.27 °N | 2414 | 2007 |
| Hexi, Lanping Co., YN | HX | 10 | East of Lancang River | 99.38 °E | 26.86 °N | 2511 | 2009 |
| Tongdian, Lanping Co., YN | TD | 7 | East of Lancang River | 99.53 °E | 26.69 °N | 2532 | 2009 |
| Jianchuan Co., YN | JCH | 20 | South of Middle Jinsha River | 99.90 °E | 26.40 °N | 2521 | 2009 |
| Hutiaoxia, Shangeri-La Co., YN | XGLL | 10 | North of Upper Jinsha River | 100.07 °E | 27.11 °N | 2002 | 2011 |
| Ma’anshan, Weishan Co., YN | WSH | 9 | Upper Red River | 100.14 °E | 25.34 °N | 2030 | 2009 |
| Baihanchang, Lijiang, YN | LJ | 7 | South of Middle Jinsha River | 100.24 °E | 26.87 °N | 2182 | 2009 |
| Songgui, Heqing Co., YN | HQ | 6 | South of Middle Jinsha River | 100.20 °E | 26.35 °N | 1944 | 2011 |
| Jizushan, Binchuan Co., YN | BCH | 3 | South of Middle Jinsha River | 100.40 °E | 25.94 °N | 1860 | 2011 |
| Nanjian Co., YN | NJ | 10 | Upper Red River | 100.49 °E | 24.90 °N | 2187 | 2009 |
| Yongbei, Yongsheng Co., YN | YSH | 9 | North of Middle Jinsha River | 100.75 °E | 26.69 °N | 2150 | 2007 |
| Xibuhe, Ninglang Co., YN | NL | 10 | North of Middle Jinsha River | 100.79 °E | 27.00 °N | 2203 | 2011 |
| Wudingshan, Nanhua Co., YN | NH | 9 | Upper Red River | 100.81 °E | 24.97 °N | 2079 | 2009 |
| Rongjiang, Huaping Co., YN | HP | 4 | North of Middle Jinsha River | 101.28 °E | 26.57 °N | 1458 | 2007 |
| Fapiao,Shuangbai Co., YN | SHB | 9 | South of Middle Jinsha River | 101.85 °E | 24.61 °N | 2081 | 2009 |
| Sanyingpan, Luquan Co., YN | LQ | 2 | South of Middle Jinsha River | 102.52 °E | 25.98 °N | 2197 | 2009 |
| Haiba, Huidong Co., SCH | HD | 11 | North of Middle Jinsha River | 102.70 °E | 26.57 °N | 2366 | 2007 |
| Songming Co., YN | SM | 8 | South of Middle Jinsha River | 102.81 °E | 25.37 °N | 2151 | 2009 |
| Dabanqiao, Kunming, YN | KM | 10 | South of Middle Jinsha River | 102.86 °E | 25.07 °N | 2031 | 2009 |
| Jinzhong, Huize Co., YN | HZ | 9 | South of Middle Jinsha River | 103.51 °E | 26.00 °N | 2091 | 2007 |

N = numbers of strains with four gene markers sequenced.

Long. = Longitude

Lat. = Latitude

Alt. = Altitude

Col. Year = Collection Year

Table S2 Details of gene markers and relative primers used in this study

| **Gene marker** | **Position in genome of *Tuber melanosporum*** | **Primers** |
|---|---|---|
| **Tic1** | Scaffold_45<br>420308-420732 | F: ACATAAGTCATCGCCGAAGC<br>R: TAACAATTCGCACGTGATGC |
| **Tic2** | Scaffold_29<br>875345-875844 | F: CAACATCATGGCGAACTGAG<br>R: ATCTCTCACCAGGGATCACG |
| **Tic3** | Scaffold_3<br>1441293-1441766 | F: GCCTCAGTCATCTCCATCCT<br>R: ACCGACGTTGACAACACCAT |
| ***mcm*7** | Scaffold_10<br>107241-107776 | F: ACGCGSGTGTCRGAWGTYAAGCC<br>Tic_F[#]: ACCCGTGTGTCGGACGTCAAGCC<br>R: GATTTKGCGACGCCGGGRTCTCCCAT |

[#] Designed for sequencing purpose.

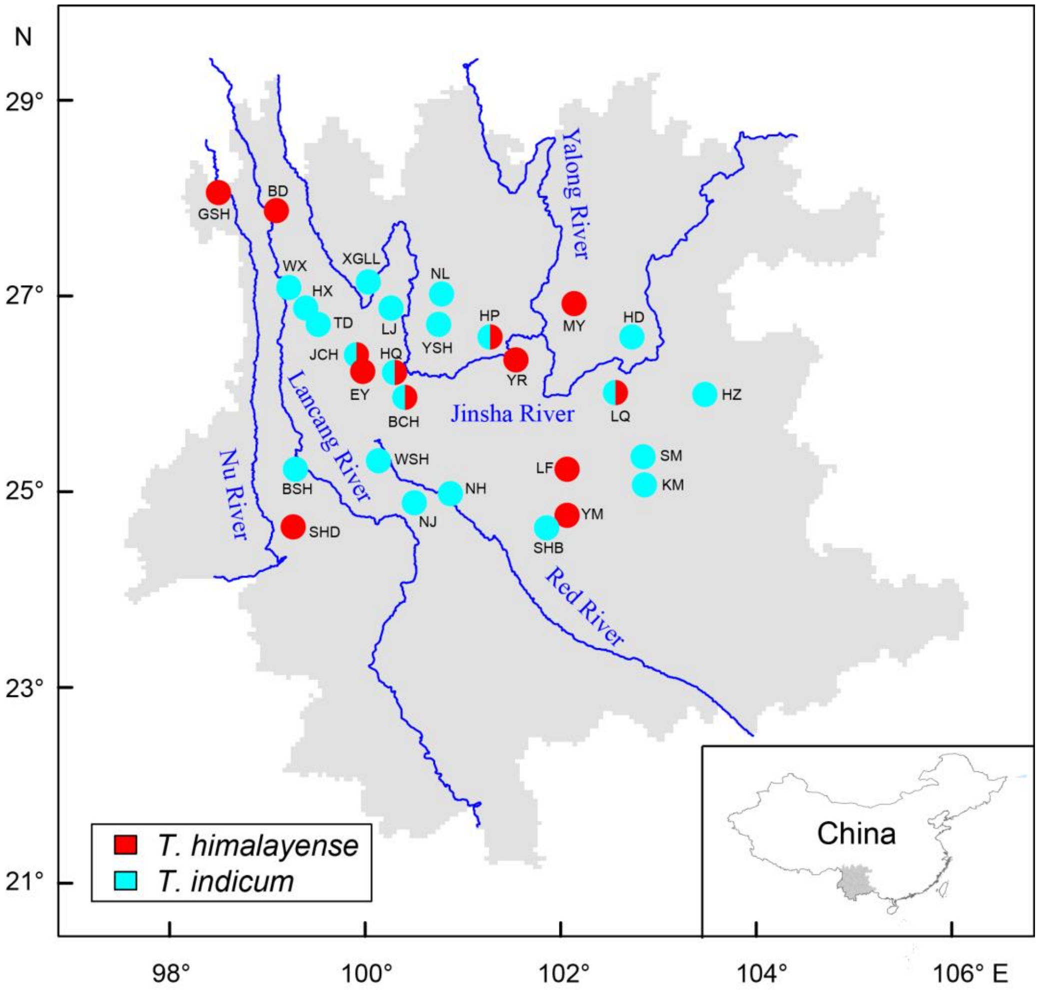


Fig. S1 A map showing the collection sites for the *Tuber indicum* species complex

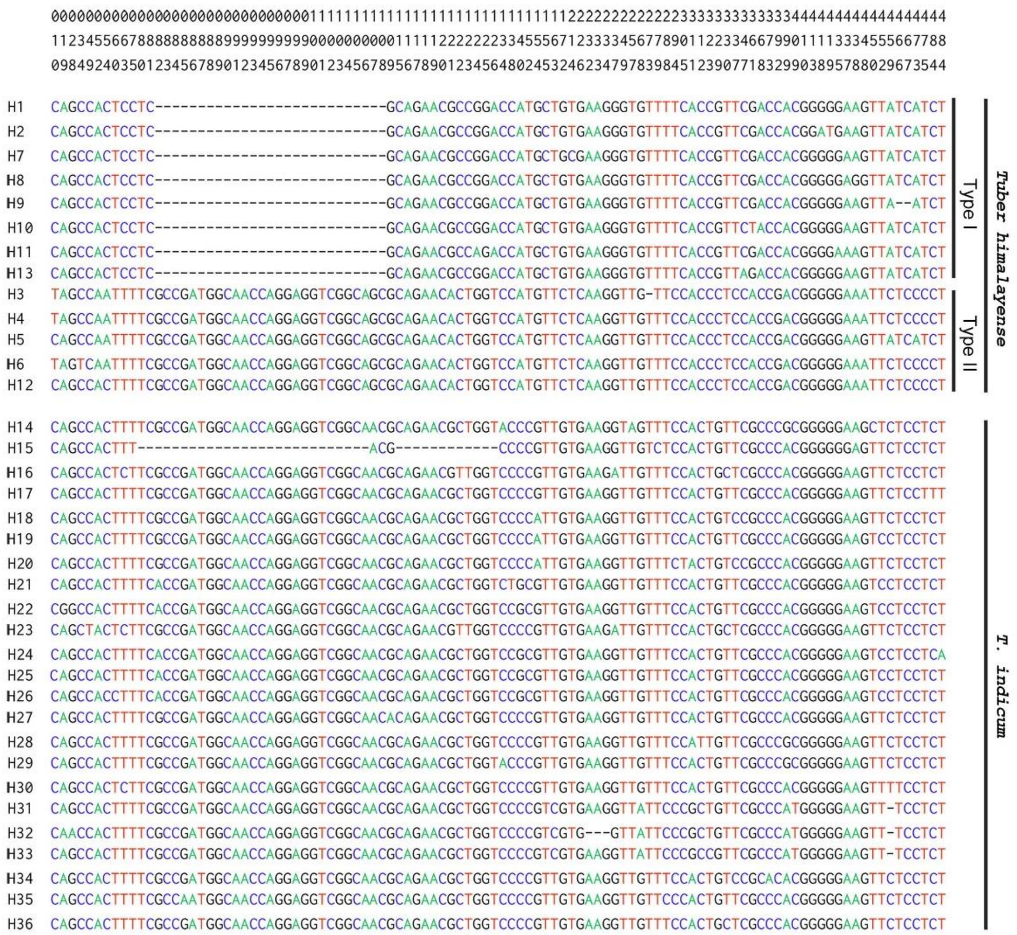


Fig. S2 Sequence variations of Tic3 in *T. indicum* and *T. himalayense*
